# Supplementary material for: Modeling glioblastoma heterogeneity as a dynamic network of cell states
Source: Mol Syst Biol. 2021 Sep 16;17(9):e10105. doi: 10.15252/msb.202010105 (PMC8444284; doi:10.15252/msb.202010105)
Supplement: Supplementary file 5 — Source Data for Figure 3 [file MSB-17-e10105-s001.zip › Figure3A_sourcedata/GSEA_3065/nosigdb_state3.GseaPreranked.1622718426028/NB_KI_CLUSTER1.html]

Details for gene set NB\_KI\_CLUSTER1[GSEA]

|  || Dataset | state3 |
| Phenotype | NoPhenotypeAvailable |
| Upregulated in class | na\_neg |
| GeneSet | NB\_KI\_CLUSTER1 |
| Enrichment Score (ES) | -0.9507466 |
| Normalized Enrichment Score (NES) | -2.8000915 |
| Nominal p-value | 0.0 |
| FDR q-value | 0.0 |
| FWER p-Value | 0.0 |
Table: GSEA Results Summary

  

Fig 1: Enrichment plot: NB\_KI\_CLUSTER1      
 Profile of the Running ES Score & Positions of GeneSet Members on the Rank Ordered List

  

| PROBE | GENE SYMBOL | GENE\_TITLE | RANK IN GENE LIST | RANK METRIC SCORE | RUNNING ES | CORE ENRICHMENT || 1 | RPL3 |  |  | 8247 | -0.238 | -0.9303 | No |
| 2 | RPL10 |  |  | 8426 | -0.304 | -0.9311 | Yes |
| 3 | RPS29 |  |  | 8446 | -0.313 | -0.9130 | Yes |
| 4 | RPL13A |  |  | 8472 | -0.325 | -0.8948 | Yes |
| 5 | RPS7 |  |  | 8486 | -0.333 | -0.8748 | Yes |
| 6 | RPL27A |  |  | 8541 | -0.358 | -0.8579 | Yes |
| 7 | RPS4X |  |  | 8542 | -0.359 | -0.8346 | Yes |
| 8 | RPL21 |  |  | 8588 | -0.393 | -0.8144 | Yes |
| 9 | RPS3 |  |  | 8598 | -0.400 | -0.7896 | Yes |
| 10 | RPS27A |  |  | 8620 | -0.422 | -0.7647 | Yes |
| 11 | RPS27 |  |  | 8638 | -0.441 | -0.7381 | Yes |
| 12 | RPL39 |  |  | 8640 | -0.443 | -0.7096 | Yes |
| 13 | EEF1A1 |  |  | 8646 | -0.450 | -0.6811 | Yes |
| 14 | RPS19 |  |  | 8648 | -0.453 | -0.6519 | Yes |
| 15 | RPL23A |  |  | 8649 | -0.454 | -0.6225 | Yes |
| 16 | RPS18 |  |  | 8658 | -0.465 | -0.5933 | Yes |
| 17 | RPS25 |  |  | 8661 | -0.467 | -0.5634 | Yes |
| 18 | RPL34 |  |  | 8671 | -0.476 | -0.5336 | Yes |
| 19 | RPS3A |  |  | 8682 | -0.495 | -0.5027 | Yes |
| 20 | RPL13 |  |  | 8685 | -0.499 | -0.4707 | Yes |
| 21 | RPLP2 |  |  | 8697 | -0.515 | -0.4387 | Yes |
| 22 | RPL18A |  |  | 8707 | -0.532 | -0.4053 | Yes |
| 23 | RPL32 |  |  | 8715 | -0.556 | -0.3702 | Yes |
| 24 | RPS6 |  |  | 8718 | -0.559 | -0.3342 | Yes |
| 25 | RPS15A |  |  | 8719 | -0.560 | -0.2980 | Yes |
| 26 | RPL28 |  |  | 8727 | -0.604 | -0.2598 | Yes |
| 27 | RPLP1 |  |  | 8728 | -0.606 | -0.2206 | Yes |
| 28 | RPS8 |  |  | 8730 | -0.621 | -0.1806 | Yes |
| 29 | TPT1 |  |  | 8735 | -0.644 | -0.1394 | Yes |
| 30 | RPS2 |  |  | 8739 | -0.668 | -0.0965 | Yes |
| 31 | RPL41 |  |  | 8743 | -0.718 | -0.0504 | Yes |
| 32 | RPS12 |  |  | 8747 | -0.792 | 0.0005 | Yes |
Table: GSEA details [plain text format]

  

Fig 2: NB\_KI\_CLUSTER1: Random ES distribution      
 Gene set null distribution of ES for **NB\_KI\_CLUSTER1**
